# When Malaria Slips a Vaccine's Net

**DOI:** 10.1371/journal.pbio.1001370

**Published:** 2012-07-31

**Authors:** Caitlin Sedwick

**Affiliations:** Freelance Science Writer, San Diego, California, United States of America

**Figure:**
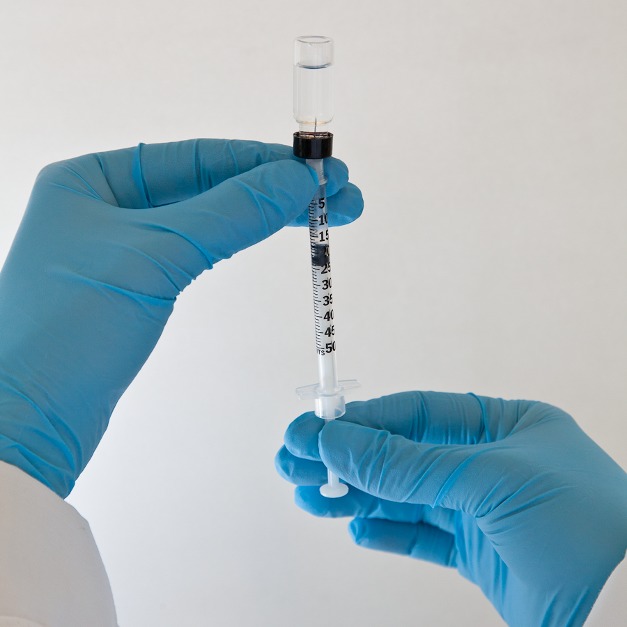
The hypothesis that some vaccines might promote the evolution of virulent pathogens is tested using a blood-stage vaccine against rodent malaria. Image credit: Roel Fleuren.

*Plasmodium falciparum* is a protozoan parasite that, along with its close relatives, causes malaria in humans. The organism is remarkably adaptable, quickly evolving resistance to the measures humans have deployed against it; it took the parasite only a decade to evolve resistance to chloroquine, a preventative agent first introduced in 1947. Newer drugs have fared no better.

A major push is currently being made to develop vaccines against the parasite. But here, too, the organism's adaptability works against us. Many malarial vaccines work by exposing people to a single purified parasite protein, causing people to develop antibodies that bind to the protein. Such vaccines may be “leaky”; they may not lead to complete elimination of the parasite from every vaccinated person, instead only reducing disease severity or slowing its spread to new hosts. Because the parasite mutates frequently, one major concern is that mutations in the targeted protein might let the parasite escape vaccine control (the technical term for this is “epitope evolution”). Unfortunately, as Victoria Barclay, Andrew Read, and colleagues describe in this week's *PLoS Biology*, this may not be the only problem vaccine designers should be considering.

For several years, Read's group has studied the possibility that, under pressure from leaky vaccines, malarial parasites with mutations or variations in proteins other than the targeted protein might be favored. For example, protein variants that speed up parasite replication or improve invasion of host cells could counter the effects of vaccine repression. Such protein variants would also make the parasite more virulent—that is, able to cause more severe disease symptoms or higher death rates in non-vaccinated individuals. However, until now no one had tested any actual vaccine for the possibility that it could provoke evolution of increased virulence. This is what prompted Barclay and colleagues to explore whether this can be observed in mice immunized with a vaccine based on the malarial protein AMA-1.

AMA-1-based vaccines are currently being developed for use in humans, so Barclay et al. wondered, would the process of vaccination also ultimately promote the survival or emergence of more virulent strains of the parasite? To obtain virulent parasites, the researchers followed a protocol where normal (non-virulent) parasites are allowed to grow in a mouse, extracted, and then injected into a new mouse. When this process is repeated several times (in laboratory parlance, the parasites are repeatedly “passaged” through new mouse hosts) it eventually produces virulent parasites. Using virulent parasites generated in this manner, Barclay and colleagues showed that AMA-1-vaccinated mice were less well able to control virulent than non-virulent parasites.

Passaging parasites through non-vaccinated mice therefore creates virulent parasites that can't be controlled very well by the vaccine. If leaky vaccines place additional evolutionary pressure on the parasite, it follows that passaging parasites through vaccinated mice might produce even more virulent parasites. The authors set out to test this hypothesis.

Initially, the vaccine strongly repressed the parasites' ability to survive in mice, sometimes working so well that the researchers couldn't recover enough parasites to do another round of infections. However, in a few cases the parasites did survive, and after several passages through vaccinated mice, these parasites started growing better and better in vaccinated animals. Eventually, they grew so well that they were almost as dense in their hosts' blood as were parasites growing in non-vaccinated mice. What's more, the data show that parasites passaged through vaccinated animals induce worse anemia in non-vaccinated hosts than do parasites that had been passaged through non-vaccinated mice. This was true even though there was no evidence of epitope evolution in the parasites' AMA-1 protein, suggesting virulence evolution caused the observed effects.

Whether increased virulence comes about due to accelerated parasite growth rates or other means is not clear. Regardless, the adapted parasites' higher density in host blood might make it easier to transmit them to a new host—especially in the wild, where mosquitoes substitute for laboratory needles. And because not every member of a given population will necessarily be vaccinated, increased virulence could be bad news for non-vaccinated individuals.

The idea that vaccination can inadvertently provoke the evolution of more virulent parasites is alarming. However, the disease that the rodent malarial parasite *P. chabaudi* causes in mice is very different from the one caused by its cousins in humans. As Barclay and colleagues discuss, this and other caveats make it difficult to generalize these results to field deployments of malarial vaccines in humans. The authors therefore recommend that all stage III malarial vaccine trials test parasite density and parasite transmission rates, and analyze parasites for mutations that improve transmission. This should show to what extent virulence evolution poses a problem for malarial vaccines—or other leaky vaccines—in humans.

**Barclay VC, Sim D, Chan BHK, Nell LA, Rabaa MA, et al. (2012) The Evolutionary Consequences of Blood-Stage Vaccination on the Rodent Malaria** ***Plasmodium chabaudi*** **. doi:10.1371/journal.pbio.1001368**

